# In Vitro Investigation into the Effect of Cryopreservation on the Mechanical Characteristics of Dental Hard Tissues

**DOI:** 10.3390/jfb14110551

**Published:** 2023-11-17

**Authors:** Noëmi M. C. De Roo, Kaat Toulouse, Laurent A. M. Thierens, Silke Henry, Stefanie De Buyser, Liesbeth Temmerman, Ronald M. H. Verbeeck, Guy A. M. De Pauw

**Affiliations:** 1 Oral Health Sciences, Department of Orthodontics, Ghent University, Corneel Heymanslaan 10, 9000 Ghent, Belgiumlaurent.thierens@ugent.be (L.A.M.T.); guy.depauw@ugent.be (G.A.M.D.P.); 2 Translational Neurosciences, Department of Cranio-Maxillofacial Surgery, University of Antwerp, Drie Eikenstraat 655, 2650 Edegem, Belgium; 3 Laboratory of Pharmaceutical Technology, Ghent University, Ottergemsesteenweg 460, 9000 Ghent, Belgium; 4 Biostatistics Unit, Faculty of Medicine and Health Sciences, Ghent University, C. Heymanslaan 10, 9000 Ghent, Belgium; stefanie.debuyser@ugent.be; 5 Biomaterials Group, Department of Basic Medical Sciences, Ghent University, C. Heymanslaan 10, 9000 Ghent, Belgium

**Keywords:** teeth, cryopreservation, mechanical properties, fracture, scanning electron microscope

## Abstract

Previous research has reported on hidden damage within the dentin introduced by cryopreservation, but the effect on the mechanical properties of the hard tissues at tooth level remains unclear. The main objective of this study is to investigate the effect of cryopreservation on the mechanical properties of teeth. A matched sample of 234 premolars of 117 children (9 ≤ age ≤ 16 years), bilaterally extracted for orthodontic reasons, were included. For each child, one tooth was randomly allocated to the cryopreservation group and the contralateral tooth was assigned to the control group. Static compression tests were performed to determine load to failure, stiffness, and toughness. In a subgroup of 20 teeth, a cyclic preloading or chewing simulation was performed. Additionally, the fracture mode was determined, and the microstructure of the fractured surfaces was examined using a scanning electron microscope (SEM). Linear mixed model analyses could not detect a statistical difference in the mean load to failure (*p* = 0.549), mean toughness (*p* = 0.968), or mean stiffness (*p* = 0.150) between cryopreserved and non-cryopreserved teeth. No significant difference in load to failure after cyclic preloading was detected between groups (*p* = 0.734). SEM analysis revealed comparable fracture characteristics between groups. It is concluded that cryopreservation does not affect the mean load to failure, stiffness, or toughness of teeth, indicating that hidden damage in the dentin is not critical at tooth level.

## 1. Introduction

Autologous tooth transplantation (autotransplantation) is a well-established treatment modality for tooth replacement in cases of trauma, caries, tooth impaction, or agenesis. Cryopreservation is a reversible process that ceases all biological functions of living cells or tissues at an ultra-low temperature (−196 °C).

Cryopreservation extends the scope of treatment in cases where autotransplantation cannot be carried out immediately or makes it possible for the teeth to be preserved for other purposes (such as stem cell collection). In the case of autotransplantation, it is important that not only the biological but also the physical properties of the different types of dental tissues are preserved. In vitro research reported no detrimental effects of cryopreservation on the periodontal ligament cells or pulp cells, whilst only a few studies have investigated the effect of cryopreservation on dental hard tissues. It has been suggested that the freezing process might lead to crack formation in the dental hard tissues due to the difference in thermal expansion coefficients of enamel and dentin [1,2,3].

Previous research has demonstrated that cryopreservation does not affect dentin permeability [4], dentin bond strength [5] or enamel bond strength [6]. Although the Vickers test demonstrated that the hardness of enamel was not affected by cryopreservation, a longitudinal fracture was found in ¼ of the teeth after freezing [7]. Kuhl et al. concluded that the cryopreservation of teeth did not generate cracks more than 0.8 μm wide and that the presence of prominent cracks can be related to the use of forceps during extraction [8]. 

Recently, Yan et al. [9] studied the effect of cryopreservation on the structural integrity of dentin cross-sections taken from the midcoronal portion of 73 human third molars, comparing three groups (cryopreserved, non-cryopreserved, and dimethyl sulfoxide (DMSO) treated). There was no significant difference in the elastic modulus or flexural strength between cryopreserved and control teeth. Their main conclusion was that hidden damage within the dentin introduced by cryopreservation could possibly render teeth more susceptible to mechanical failures by fatigue and fracture. The study by Angermair et al. [3] investigated the effect of cryopreservation on the fracture behaviour of 20 third molars by comparing two different storage temperatures (−80 °C versus −196 °C). Prior to the determination of the load to failure, a cyclic preloading in a chewing simulator was performed to induce a certain level of fatigue. Angermair et al. concluded that the load to failure did not differ after cryopreservation for both storage conditions. Finally, the recent study by Xu et al. [10] observed that a decrease in the elastic modulus of dentin was inversely proportional to the increase in the freezing time and the age of the teeth, whilst the compressive strength of dentin was not significantly different between cryopreserved and non-cryopreserved teeth.

The prevailing literature has mainly focused on the testing of dental dentin beams derived from third molars [4,5,9,10] and has often lacked a control group without cryopreservation [3,8]. It remains unclear how the suggested ‘hidden damage’ in dentin may affect the mechanical properties at tooth level. Teeth have a complex hierarchical structure and composition; the enamel prism arrangement at right angles to the dentin–enamel junction plays an important role on the dissipation of energy under contact loading [11]. To improve clinical relevance, it is interesting to complement the existing research on dentin with investigations at tooth level. For this reason, the objective of the present study is to investigate the effect of cryopreservation on the mechanical properties of teeth such as load to failure, stiffness, and toughness. In addition, the fracture characteristics of the enamel and dentin were investigated by microscopic evaluation of the fracture surfaces.

## 2. Materials and Methods

### 2.1. Sample Selection 

**Figure 1:**
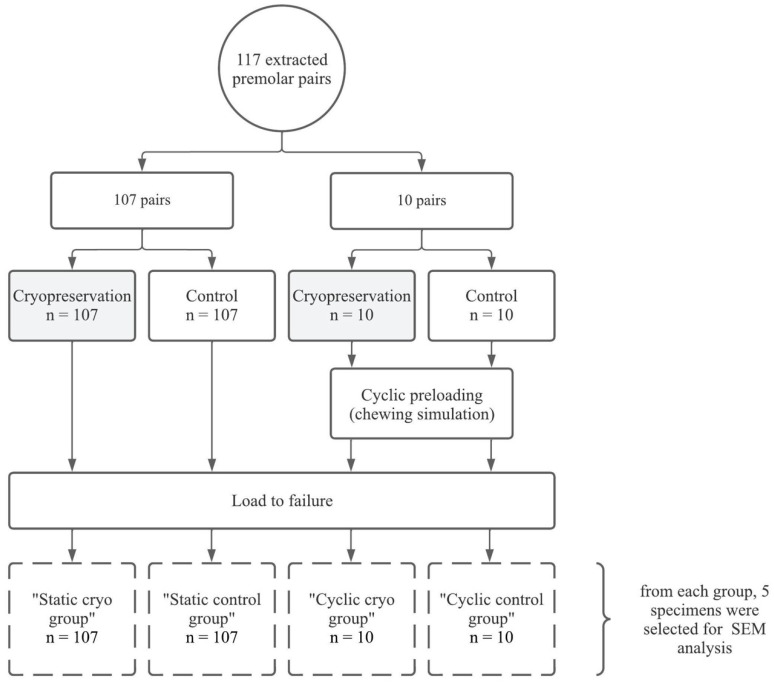
Schematic representation of the sample and test groups.

Figure 1 presents a schematic overview of the sample and test groups. A total of 234 intact premolars of 117 healthy children between the ages of 9 and 16, bilaterally extracted as part of orthodontic treatment, were included. For each child, one tooth was allocated to the cryopreservation group and the contralateral tooth was assigned to the (non-cryopreserved) control group. The study was approved by the Ethical Committee of Ghent University Hospital under the Belgian registration number GK2019-1238 and all participants signed a written informed consent form.

### 2.2. Sample Preparation

After extraction, the premolars were immediately stored in a plastic container (70 mm high × 35 mm diameter) with 20 mL of phosphate-buffered saline (PBS) at room temperature. To remove gum and tartar residues and the present biofilm, an initial cleaning was performed with hand instruments (Hu Friedy^®^, Chicago, IL, USA; scaler SM11/12A6), followed by a second cleaning with pumice stone (Equator lab supplies, Belgica Dental BV, Ghent, Belgium; pumice stone fine) using a rotating polishing brush (Henry Schein^®^, Melville, NY, USA; Prophy Brushes, Nylon) at a speed of 2500 rpm for ten seconds. Finally, the premolars were rinsed with PBS for 5 s and stored in a 0.5% chloramine solution for one week in order to disinfectthem.

After cleaning and disinfection, the teeth were visually inspected with professional loupe glasses (ExamVision, Samsø, Denmark; ICON) under 2.8× magnification and illumination (6500 K LED lamp, ExamVision, Samsø, Denmark; Focus Bright). Teeth showing anomalies, caries, restorations, wear, or other defects (e.g., fluorosis) were excluded from the study. Subsequently the crown width (mesiodistal dimension) and crown length (buccolingual/palatal dimensions) of the tooth were measured using a digital calliper with an accuracy of 0.01 mm. 

From each patient, one tooth was transported to the tooth bank for cryopreservation for two weeks. Thereafter, all teeth were embedded in polymethylmetacrylate (PMMA) (Dentaurum, Pforzheim, Germany, Orthocryl) along their longitudinal axis to secure the tooth and simulate alveolar bone support. PMMA was processed according to the manufacturer’s instructions and poured into a silicone mould (31 mm height × 15 mm diameter). The teeth were manually embedded up to 2 mm below the cementoenamel junction. To keep the teeth hydrated and to allow the further curing of the PMMA, the moulds were placed upside down in a water reservoir after ten minutes. After full curing, the PMMA samples were milled to the dimensions of the retainer used for the compression test and placed in a covered plastic container with 20 mL PBS.

### 2.3. Cryopreservation

The established tooth cryopreservation protocol of the HIRUZ (Health, Innovation and Research Institute of Ghent University Hospital) biobank was followed [12]. The teeth were saturated with DMSO by four consecutive soakings in a solution containing 2.5, 5.0, 7.5, and 10 vol% DMSO. The basic medium contained 125 mL Dulbecco’s modified Eagle’s medium (DMEM), 15 mL fetal calf serum (FCS), and 1.5 mL antibiotics/antimycotics. The teeth were frozen at a slow freezing rate of −1 °C/min from room temperature to −80 °C, plunged in liquid nitrogen and stored in isothermal freezers (−196 °C). After two weeks the teeth were rapidly thawed for 3 min in a 37 °C warm water bath. DMSO was gradually leached out by successive extractions in a solution of PBS with gradually lower concentrations of DMSO (from 10 vol%, 7.5 vol%, 5.0 vol%, and 2.5 vol% to 0 vol%). Finally, the teeth were transported in PBS to the laboratory for testing.

### 2.4. Compression Test

Static compression tests were performed using a universal testing machine (TA. HD PlusC Texture Analyser, Stable Micro Systems, Godalming, Surrey, UK). The test samples were fixed in a custom-made retainer on a heavy-duty stainless-steel platform. An axial compressive loading was applied at a crosshead speed of 0.5 mm/min. The force was applied with a custom-made cylindrical-ended steel compressive head with a diameter of 6 mm. The load to failure (N), stiffness (N/m) and toughness (N·m) were measured with a 500 kgf load cell and force–distance diagrams were generated. 

A small subgroup of 20 teeth (Figure 1), i.e., 10 tooth pairs of which one tooth was cryopreserved and the contralateral tooth was allocated to a non-cryopreserved control group, was subjected to a 50 N vertical cyclic load (50 Hz, 50,000 cycles) to induce a certain level of fatigue, prior to the determination of the load to failure [3]. Cyclic loading was applied with the tooth submerged in a bath of PBS at room temperature to prevent dehydration of the samples during testing. 

### 2.5. Data Analysis

Exponent Connect (Stable Micro Systems, UK) was used to record the raw distance (mm) and force (N) data during the compression tests. For each tooth, a force–distance diagram was plotted using MATHLAB 9_11 and the load to failure, stiffness (E-modulus) and toughness were calculated. Two observers interpreted the diagrams, and in case of doubt the fracture points were determined in agreement. The data were labelled as ‘clear measurements’ when the fracture was unambiguous and labelled as ‘subjective measurement’ when the observers defined the fracture point in mutual consultation. If no agreement was reached, the data were excluded.

**Figure 2:**
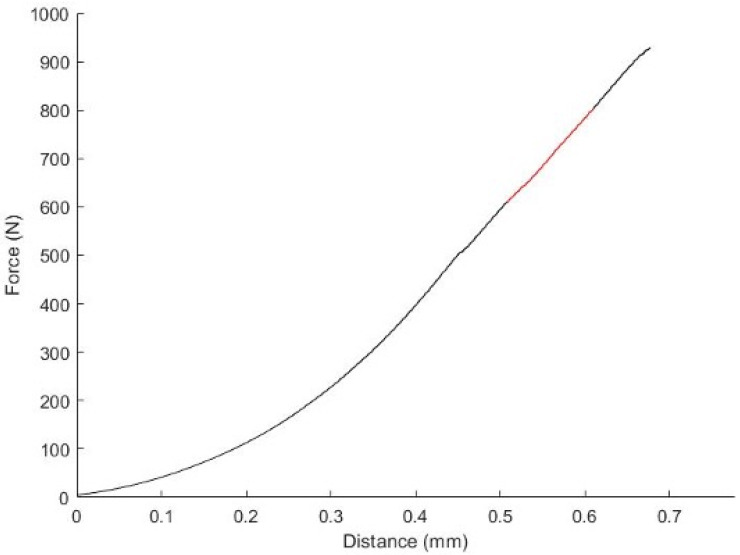
Force–distance diagram: the stiffness was calculated as the slope of the linear part of the force–distance curve before fracture, as indicated in red.

The load to failure was defined as the greatest force a material could withstand without breaking. The stiffness was calculated as the slope of the linear part of the force–distance curve before fracture (Figure 2). The toughness represents the area under the force–distance diagram.

### 2.6. Fracture Mode and SEM Analysis 

The fracture mode was defined as ‘enamel fracture’ when the fracture occurred in the enamel only, or as a ‘complex fracture’ in case of the fracture of a larger fragment including dentin or a fracture at the dentin–enamel junction. Twenty specimens were randomly and equally selected from each test group (Figure 1). The fracture surfaces were examined using a Quanta 200 FEG MKII scanning electron microscope (FEI, Hillsboro, OR, USA). Prior to analysis, the specimens were dried and a gold coating of approximately 35 nm was applied with a SC7620 Sputter Coater (Emitech, Fall River, MA, USA). Images were obtained with an accelerating voltage of 15 kV and with progressive magnification of ×2000, ×4000, ×8000, ×16,000. 

### 2.7. Statistical Analysis 

Analyses were performed with the software package R version 4.1.3. Linear mixed models were fitted for load to failure (log-transformed), toughness (log-transformed) and stiffness using the R package “Ime4” and a random factor for child was included to allow for a within-child correlation. The main models contained ‘group’ (cryopreserved versus non-cryopreserved), ‘crown length’ and ‘crown width’ in the fixed effects part. Sensitivity analyses included only observations labelled as clear measurements. Exploratory analyses compared cryopreservation with non-cryopreservation in subgroups according to tooth type (maxilla versus mandibula) or root development (open versus closed apex). The underlying models allowed for all two-way interactions with tooth type or root development.

The estimated marginal means with 95% confidence interval (CI) were computed, as well as the mean ratio (load to failure and toughness) or mean difference (stiffness) of the comparison cryopreservation versus non-cryopreservation with corresponding 95% CI and *p*-value. Non-inferiority of cryopreservation versus non-cryopreservation with respect to mean load to failure, toughness or stiffness was concluded if the lower limit of the 95% CI of the mean ratio was greater than or equal to a predefined non-inferiority margin (NIM). A difference of 10% in mechanical characteristics between the cryopreserved and control group was considered clinically relevant, e.g., the NIM was set at 0.90 for the mean ratio of load to failure and toughness and was set at −174 N/m for the mean difference in stiffness. 

The non-inferiority of cryopreservation versus non-cryopreservation at an individual tooth pair level was assessed via the proportion of tooth pairs where the (relative) difference fell above the NIM. A 95% Wilson score CI was calculated around this estimated proportion. The (relative) difference between the cryopreserved and non-cryopreserved teeth was plotted against the mean outcome within each tooth pair.

Furthermore, a multiple linear mixed model was carried out for each outcome including the main effects of ‘group’, ‘crown length’, ‘crown width’, ‘tooth type’ and ‘root development’ in the effects part (assuming no interactions).

Due to the much smaller sample size of the group which was analysed for load to failure after cyclic preloading, no distributional assumptions could be made. Hence, the comparison between the matched cryopreserved and non-cryopreserved teeth was performed using a Wilcoxon signed-rank test. The significance level was set at 0.05. 

## 3. Results

### 3.1. Descriptive Statistics 

The present study included 117 patients (52 males and 65 females) with a mean age of 13.48 years old (range 9.4 y–16.1 y). A total of 107 tooth pairs were tested for load to failure with static loading. For the other 10 tooth pairs, the load to failure was determined after cyclic preloading (Figure 1). There were 72 maxillary tooth pairs and 45 mandibular tooth pairs. Root development was complete in 82 tooth pairs, while 35 tooth pairs had an open apex. The crown dimensions and observers’ agreement on the fracture points (data labelled as clear/subjective or excluded) are presented in Table 1. 

The mean load to failure with or without cyclic preloading, mean toughness, and mean stiffness of the cryopreserved and non-cryopreserved teeth are presented in Table 2. 

### 3.2. Load to Failure 

**Figure 3:**
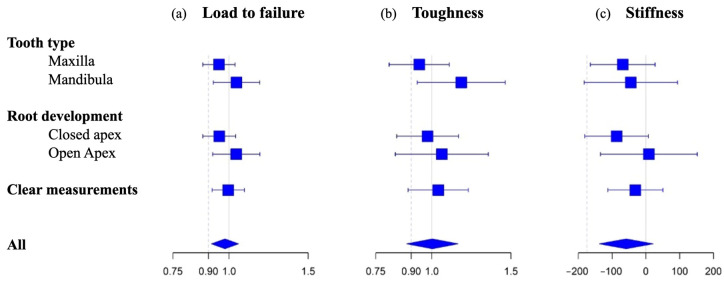
Comparison of geometric mean load to failure (**a**) and toughness (**b**) between cryopreserved teeth versus non-cryopreserved teeth. Comparison of arithmetic mean stiffness (**c**) between cryopreserved teeth versus non-cryopreserved teeth. The predefined NIM of 0.90 is indicated as a dotted line.

The marginal mean load to failure was estimated at 922 N (95% CI: 835–1018 N) in the cryopreservation group and 942 N (95% CI: 852–1040 N) in the control group. Hence, the estimated mean load to failure for cryopreserved teeth is 0.98 times the load to failure for non-cryopreserved teeth. The difference in mean load to failure between cryopreserved and non-cryopreserved teeth is not significant (adjusted mean ratio = 0.98; 95% CI: 0.91–1.05; *p* = 0.549) as illustrated by the forest plot in Figure 3. The lower limit of the 95% CI falls above the predefined NIM of 0.90, so non-inferiority can be concluded. Consequently, a statistically significant or clinically (based on the NIM) relevant difference in mean load to failure could not be detected between cryopreserved teeth and non-cryopreserved teeth. A sensitivity analysis, which only included clear measurements, led to the same conclusion of non-inferiority of the main analysis.

Figure 3a presents a comparison of the geometric mean load to failure between cryopreserved and non-cryopreserved teeth for the entire sample and by subgroups. The effect of cryopreservation was not different for maxillary or mandibular teeth (*p* = 0.219) or according to the root development (*p* = 0.242). 

**Figure 4:**
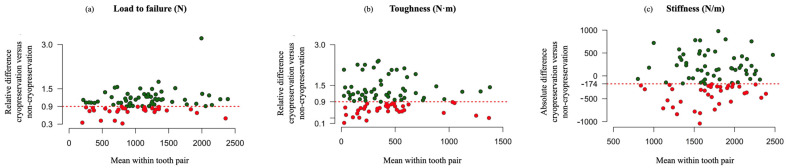
(**a**) Relative difference in cryopreservation versus non-cryopreservation in load to failure versus mean load to failure within each tooth pair. (**b**) Relative difference in cryopreservation versus non-cryopreservation in toughness (N·m) versus mean toughness within each tooth pair. The dotted red line represents the non-inferiority margin for the relative difference. (**c**) Absolute difference in cryopreservation versus non-cryopreservation in stiffness (N/m) versus mean stiffness within each tooth pair. The dotted red line represents the non-inferiority margin for the absolute difference.

In 65% of children, the cryopreserved tooth did not score lower compared to the non-cryopreserved tooth with respect to the load to failure (95% Wilson Score CI: 55–74%) (Figure 4a).

Table 3 presents the results from a multiple linear mixed model analysis, explaining which factors are significantly associated with load to failure. The mean load to failure was significantly lower (45%) in the mandibular teeth when compared to the maxillary teeth, based on the comparison of teeth within the same group (cryopreservation or control), with the same crown length, width, and maturity (adjusted mean ratio of 0.55; 95% CI: 0.43–0.69; *p* < 0.001). Also, there was a significant positive association between crown length and the mean load to failure: for every unit increase in crown length, the estimated mean load to failure increased by 17% (adjusted mean ratio = 1.17; 95% CI: 1.02–1.33; *p* = 0.023).

### 3.3. Toughness

The estimated marginal mean toughness was identical in the cryopreservation and control group, i.e., 276 N·m (95% CI: 233–328). Hence, the difference in toughness between both groups (adjusted mean ratio = 1; 95% CI: 0.88–1.14; *p* = 0.968) is statistically insignificant as illustrated in Figure 3b. The non-inferiority assessment was borderline inconclusive in both the main analysis as well as the sensitivity analysis (including only the clear measurements), with lower limits of the 95% CI for the mean ratio of 0.88 and 0.89, respectively (less than NIM of 0.9). Subgroup analyses showed that the effect of cryopreservation on toughness was not different between subgroups according to tooth type (*p* = 0.120) or root development (*p* = 0.614). 

In 61% of children, the cryopreserved tooth did not score lower than the non-cryopreserved tooth with respect to toughness (95% Wilson score CI: 50–70%) (Figure 4b).

Based on a multiple linear mixed model analysis, there was a significantly lower mean toughness in the mandibular teeth (63% less) when compared to the maxilla (adjusted mean ratio = 0.37; 95% CI: 0.25–0.57; *p* < 0.001). There was a positive significant association between crown length and mean toughness: for every unit increase in length, the estimated mean toughness increases by 27% (adjusted mean ratio = 1.27; 95% CI: 1.02–1.59; *p* = 0.038). 

### 3.4. Stiffness

The marginal mean stiffness was estimated at 1683 N/m (95% CI: 1609–1756) for the cryopreservation group and 1741 N/m (95% CI: 1667–1814) for the control group. No statistically significant nor clinically relevant difference in mean stiffness could be detected between the cryopreserved teeth and non-cryopreserved teeth (adjusted mean difference = −58 N/m; 95% CI: −137–21 N/m; *p* = 0.150). The sensitivity analysis confirms the non-inferiority (*p* = 0.449). Exploratory analyses showed no indication that the effect of cryopreservation on mean stiffness is different between subgroups according to tooth type (*p* = 0.788) or root development (*p* = 0.273). 

Figure 3c presents the forest plot of mean difference in stiffness of the cryopreserved teeth versus the non-cryopreserved teeth with a NIM of −174 N/m. 

In 60% of children, the cryopreserved tooth did not score less compared to the non-cryopreserved tooth with respect to stiffness (95% Wilson score CI: 49–69%) (Figure 4c). 

However, the multiple linear mixed model analysis showed a positive significant association between crown length and mean stiffness (adjusted mean difference = 135 N/m; 95% CI: 26–245; *p* = 0.017).

### 3.5. Load to Failure after Cyclic Preloading

The observed median load to failure [25–75th pct] after cyclic preloading was 1055.2 N [647.42 N–1328.7 N] for the cryopreserved teeth and 909.84 N [405.64 N–1350.58 N] for the non-cryopreserved teeth. Cyclic preloading did not significantly affect the load to failure according to a Wilcoxon signed-rank test (*p* = 0.734).

### 3.6. Fracture Mode 

Of the 234 teeth, 28.6% showed an enamel fracture and 71.4% showed a complex fracture in the dentin or at the dentin–enamel junction. This distribution was the same in both the cryopreserved group and non-cryopreserved group with 28.2% and 29.3% of enamel fractures, respectively. In 87.2% of the tooth pairs, the teeth had the same fracture mode. In the maxilla, most teeth had a complex fracture (95.8%), whilst in the mandibula, an enamel fracture was seen more often (67.8%). The observers’ agreement was higher on the data of the complex fractures (87.4% clear measurements) when compared to the data of the enamel fractures (50.7%). Samples that presented an enamel fracture showed a lower mean load to failure (428 N; 95% CI: 242–614) than teeth with a complex fracture (1273 N; 95% CI 786–1760).

### 3.7. SEM Analysis

**Figure 5:**
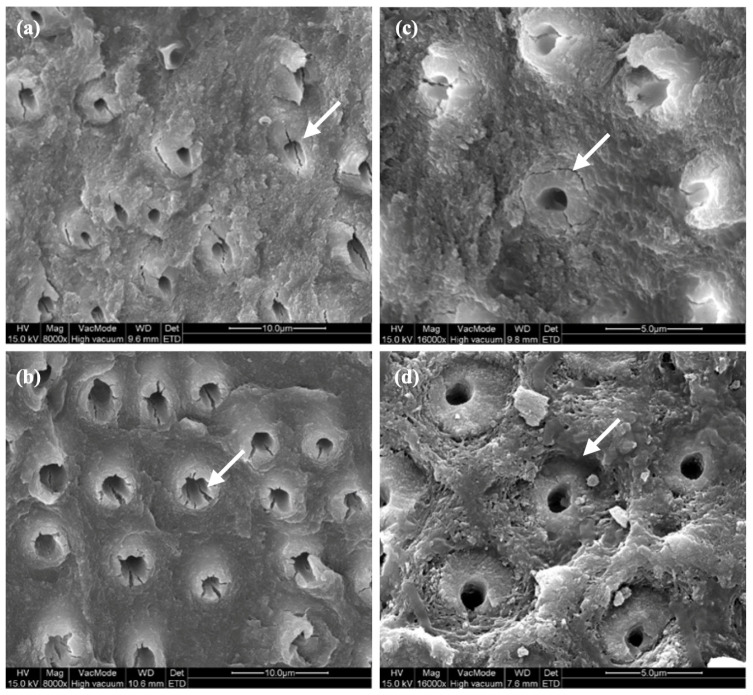
SEM images presenting peritubular cracks (**a**,**b**), a circular crack (**c**) or sunken peritubular dentin (**d**).

The selection of the 20 specimens for the SEM analysis was performed randomly and in equal numbers from the loading (with or without cyclic preloading) and test groups (cryopreservation/control). Two teeth were excluded from the cyclic control group. One specimen was excluded due to the size of the fracture surface, which was too big to fit under the microscope. The second specimen was excluded because no images could be made of the dentin tubules due to the fracture direction of the fragment. Clear radial cracks were present in the peritubular dentin of all tubuli after cryopreservation, after both static and cyclic loading (Figure 5a,b). In three samples of the cryopreserved group subjected to cyclic loading, a circular crack was found (Figure 5c). Sunken peritubular dentin was noted in only a few tubuli in a few samples but could be linked neither to the cryopreservation nor the loading protocol (Figure 5d).

## 4. Discussion

The objective of this study was to investigate the effect of cryopreservation on the mechanical properties and fracture characteristics of the hard tissues of intact premolars. The load to failure, toughness, and stiffness of premolars are apparently not affected by the standardized cryopreservation procedure used in the present study. Moreover, differences in load to failure and stiffness between cryopreserved teeth and non-cryopreserved teeth are clinically insignificant based on a non-inferiority margin of 10%. This margin was set arbitrarily as it was not possible to rely on the current literature to assess an objective NIM. The evaluation of non-inferiority with respect to mean toughness was borderline inconclusive, with a lower limit of the 95% CI that fell just below the predefined NIM of 10% (0.88 < 0.90). 

Yan et al. [9] reported no significant difference in elastic modulus or flexural strength of dentin after cryopreservation, but they detected a 20% lower fatigue strength in the cryopreserved group. Based on the latter and a microscopic examination of the microstructure of the dentin, the authors concluded that hidden damage within the dentin could render teeth more susceptible to mechanical failures by fatigue and fracture after cryopreservation [9]. In this respect, the present study also found radial peritubular cracks after cryopreservation as well as sporadically sunken peritubular dentin which, however, could not be linked to the cryopreservation nor loading protocol. Moreover, the load to failure after cyclic preloading, simulating a masticatory load on the teeth, was not significantly affected by cryopreservation.

The study by Xu et al. [10] examined the biomechanical properties and microstructural changes of the dentin of teeth as a function of biological age (a youth group aged 18–25, a middle-aged group aged 26–55, and an elderly group aged >56 years) and duration of cryopreservation (storage at −196 °C for 7, 30, and 90 days). They observed that the inner wall of the dentin tubules had increased roughness in the elderly group that was cryopreserved for 90 days. Such a change was not found for the youth group up to 30 days storage in liquid nitrogen. Consequently, for these groups the storage duration does not appear critical in first instance. The present study focusses on the teeth of young children aged 9–16 years, the choice for restricting the storage duration to two weeks appears to be justified and does not exclude a study of the effect of longer durations in the future. The focus on the teeth of young children aged 9–16 years is based on the actual clinical situation. In fact, cryopreservation is mainly applied to the teeth of young children with the intention of future replantation. In older patients, cryopreservation and autotransplantation of teeth is less frequently performed and more often ‘simpler’ tooth replacements such as dental implants are preferred. The storage duration for two weeks at −196 °C is in accordance with the studies of Yan et al. (10 days) [9] and Angermair et al. [3] (two weeks). Although it could be interesting to investigate the effect of the storage duration at −196 °C, the cooling and thawing process appear more important as these processes could lead to damage in the dental hard tissues. Such damage can be induced by the formation of ice crystals or stresses resulting from the thermal expansion/contraction mismatch of enamel and dentin. The formation of water crystals depends on multiple factors such as freezing rate, viscosity, and freezing temperature [10], so the cryopreservation conditions play an important role. A slow freezing rate has been shown to prevent crystal formation and reduce cell damage in the cryopreservation of soft tissues. Adding cryoprotectants aids this process by increasing the viscosity and decreasing the freezing temperature. As a tooth ages, the mineral fraction of peritubular dentin will increase and the lumen of the tubuli will become narrower. Small mineral islands within the tubuli may prevent the DMSO from infiltrating adequately, possibly enabling residual water to cause more damage during the freezing protocol. The present study used a four-step procedure with gradually increasing and decreasing DMSO percentages whilst in the study of Xu et al. [10] the sample was placed in a tube with 10% DMSO for 30 min.

Hence, the suggestion by Yan et al. [9] that a damage in the microstructure of dentin, that appeared to have caused a decrease in durability of the dentin beams, cannot be generalized at tooth level. In this respect the unique features of the dentin–enamel junction (DEJ) may have a healing or moderating capacity. Due to its viscoelasticity, the DEJ provides a load attenuation mechanism between the enamel and the dentin and plays an important role in the hard-tissue biomechanics [13].

It is challenging to standardize in vitro testing at tooth level. Previous research has often lacked a non-cryopreserved control group [3,8]. To minimize the biological differences and create homogeneous groups, a pairwise comparison of a cryopreserved premolar with a non-cryopreserved contralateral premolar of the same individual using a large sample of 117 tooth pairs was performed. The transport and test conditions were controlled and identical in the cryopreservation and control group. 

Another challenge was to standardize the vertical loading of the occlusal surfaces of premolars. The tooth morphology of a premolar does not always allow for an initial three-point contact on the occlusal surface when applying the vertical load balancing, especially in lower premolars where the lingual cusp is often very small. In some cases, the probe ‘slipped’ when contacting the specimens, resulting in an irregularity in the force–distance diagrams. The interpretation of the diagrams was therefore performed by two independent observers and the measurements were labelled as unclear when a discussion was needed. By performing a sensitivity analysis, using only the clear measurements, the error of the method was evaluated. There was no difference in conclusion between the cryopreserved group and the non-cryopreserved group when examining the whole sample or when evaluating only the clear measurements.

The application of cryopreservation to teeth is a small niche mostly due to the strict legislation and guidelines for management and organization of a tooth bank. Currently, mature teeth can also be successfully transplanted immediately without cryopreservation [14,15]. Cryopreservation is still applied in patients where premolars need to be extracted for orthodontic reasons and may serve to replace an incisor lost due to previous trauma. During the orthodontic preparation of the receptor zone, the tooth is transferred to the tooth bank for cryopreservation. For this reason, the present study selected premolars from patients in orthodontic treatment and a subgroup analysis based on the root development (open versus closed apices) was performed. As stated by Kuhl et al. [8] root development, e.g., the size of the apical foramen, may influence the extent to which cryoprotectants can infiltrate. When the infiltration of the cryoprotectant is insufficient, intracellular crystallization may cause expansion of the pulp tissue, resulting in crack formation in the dental hard tissues. However, the subgroup analysis showed no indications that the mechanical properties after cryopreservation are different between teeth with open or closed apices.

## 5. Conclusions

The present study demonstrates that cryopreservation does not affect a tooth’s mean load to failure, stiffness, or toughness by a clinically relevant amount, when compared to its non-cryopreserved, matched, contralateral tooth. Subgroup analyses showed no differences in effect of cryopreservation between maxillary and mandibular teeth, nor between teeth with open or closed apices. The possible effect of hidden damage in the dentin on the mechanical properties appears to be negligible at tooth level. 

## Figures and Tables

**Table 1:** Descriptive statistics of the tooth dimensions and observers’ agreement.

	Mean Crown Width (Range) in mm	Mean Crown Length (Range) in mm	Observer’s Agreement
Cryopreserved teeth	7.39 (6.30–8.70)	9.17 (7.45–11.03)	93 clear measurements 12 subjective measurements 12 excluded pairs
Non-cryopreserved teeth	7.41 (6.48–8.50)	9.15 (7.21–11.02)	87 clear measurements 18 subjective measurements 12 excluded pairs

**Table 2:** Results of the compression tests reporting the load to failure, toughness, and stiffness.

	Mean (Range) Load to Failure in N	Mean (Range) Load to Failure after Cyclic Preloading in N	Mean (Range) Toughness in N·m	Mean (Range) Stiffness in N/m
Cryopreserved teeth	1093 (104–3040)	950 (284–1592)	423 (6–2678)	1681 (734–2703)
Non-cryopreserved teeth	1074 (201–3151)	1042 (146–3068)	424 (5–2235)	1736 (640–2590)

**Table 3:** Explanatory factors in the multiple linear mixed model analysis on load to failure. Statistically significant *p*-values are indicated with *.

	Estimated Mean Ratio (95% CI)	*p*-Value
Group (control versus cryopreservation)	1.01 (0.95–1.08)	0.700
Crown length	1.17 (1.02–1.33)	0.023 *
Crown width	1.01 (0.85–1.22)	0.879
Tooth type (mandibular versus maxillary)	0.55 (0.43–0.69)	<0.001 *
Root development (open versus closed apex)	1.02 (0.85–1.22)	0.846

## Data Availability

The raw/processed data required to reproduce these findings are available on request from the corresponding author.
